# Effect of diluent type, cryoprotectant concentration, storage method and freeze/thaw rates on the post-thaw quality and fertility of cryopreserved alpaca spermatozoa

**DOI:** 10.1038/s41598-019-49203-z

**Published:** 2019-09-06

**Authors:** C. C. Stuart, J. L. Vaughan, C. M. Kershaw, S. P. de Graaf, R. Bathgate

**Affiliations:** 1 Sydney School of Veterinary Science, Faculty of Science, The University of Sydney, Sydney, NSW 2006 Australia; 2 Cria Genesis, PO Box 406, Ocean Grove, VIC 3226 Australia; 3 Department of Animal Production, Welfare and Veterinary Sciences, Harper Adams University, Shropshire, UK; 4 School of Life and Environmental Sciences, Faculty of Science, The University of Sydney, Sydney, NSW 2006 Australia

**Keywords:** Biological techniques, Physiology

## Abstract

This study compared protocols for cryopreservation of ejaculated, papain-treated alpaca spermatozoa. This included different concentrations of egg yolk (EY; 5, 10 or 15%) and glycerol (2, 5 or 10%), diluent types (SHOTOR, lactose, skim milk or INRA-96™), freeze rates (2, 4 or 8 cm above liquid nitrogen; LN), thaw rates (37 °C for 1 min or 42 °C for 20 sec) and storage vessels (pellets, 0.25 mL straws or 0.5 mL straws). Spermatozoa were assessed pre-freeze and 0, 30, 60 and 90 min post-thaw. Forty-one hembras were inseminated with either fresh, papain-treated or frozen-thawed spermatozoa. Motility was affected by EY concentration (P < 0.001), diluent type (P < 0.001), freeze rate (P = 0.003) and storage vessel (P = 0.001). Viability was affected by EY concentration (P < 0.001), diluent type (P < 0.001), storage vessel (P = 0.002) and thaw rate (P = 0.03). For artificial insemination (AI), semen was diluted 1:3 in a lactose-based diluent, with 5% EY and glycerol. Freezing was in 0.5 mL straws, 2 cm above LN for 4 min then thawing at 37 °C for 1 min. Pregnancy rates of those ovulated (n = 26) were not different (1/5 fresh, 1/4 papain-treated, 0/17 frozen-thawed; P = 0.10). Pregnancy can be achieved after AI with papain-treated spermatozoa. Further work is needed to determine the optimal dose, timing and location for insemination.

## Introduction

Semen cryopreservation is commonly used in livestock breeding programs, as it allows superior male genetics to be easily distributed, facilitating a greater rate of genetic gain for desirable traits within and between herds when used in conjunction with artificial insemination (AI). In alpacas and other camelids, these technologies are not well-developed, in part due to the unique semen characteristics of these species. Alpaca semen has low sperm concentration and motility, as well as viscous seminal plasma that makes diluting and processing difficult^1^. The viscous nature of the seminal plasma prevents cryoprotective agents, such as egg yolk and glycerol from coming into contact with the sperm plasma membrane. Kershaw-Young and Maxwell^2^ found that the viscosity in alpaca seminal plasma is caused by the protein mucin 5B and that by adding the protease papain to alpaca semen, viscosity could be reduced to zero, without adversely affecting the motility, acrosome integrity, viability and DNA integrity of the spermatozoa^3^. This discovery has facilitated renewed attempts to cryopreserve alpaca semen.

A number of cryoprotectants and diluent types have been tested for cryopreservation of alpaca spermatozoa. Morton, *et al*.^1^ diluted epididymal alpaca spermatozoa (to bypass the issue of viscous seminal plasma) with citrate- Tris- and lactose-based diluents and froze in straws and pellets. They found that a lactose-based diluent, combined with freezing in straws rather than pellets was most effective at retaining acrosome integrity and motility post-thaw. However, Kershaw-Young and Maxwell^4^ found that a Tris-citrate-fructose diluent preserved ejaculated, papain-treated sperm motility during freezing and thawing better than a lactose-based diluent, though the post-thaw motility was low. Other studies have found skim milk diluents yielded higher post-thaw survival than Tris-based diluents^5,6^. In Experiment 3, four semen diluents were compared as potential cryodiluents for papain-treated alpaca spermatozoa in order to clarify these conflicting results.

Commonly used non-penetrating cryoprotectants include whole egg yolk, low-density lipoproteins derived from egg yolk^7,8^ and soy lecithin^9^. The concentration of egg yolk required to impart protection varies greatly between species as susceptibility to lipid-phase transitions and freezability has been shown to be species specific^10^. There are no studies on the effects of egg yolk concentration on alpaca spermatozoa, instead freezing studies have reported using cooling diluents supplemented with anything from 5%^5^ to 20%^11^ egg yolk, with varying success. Penetrating cryoprotectants include glycerol, dimethyl sulfoxide (DMSO) and ethylene glycol, among others, although glycerol is the most commonly used. Glycerol is a highly effective cryoprotectant, but is somewhat toxic to spermatozoa, and the extent of toxicity varies between species^12^. DMSO^13^ and ethylene glycol^5^ have been used in alpaca and llama semen freezing studies, but glycerol remains the most commonly used and concentrations between 2 and 7.5% have been studied^11,14^. Additionally, while one study found no difference between post-thaw motility and acrosome integrity when 2, 3 or 4% glycerol was used^11^, another found that 7% glycerol produced superior motility and sperm viability when compared with concentrations under 4%^14^. In Experiment 1 and 2 of the current study we aimed to test a wider range of glycerol (2, 5 or 10%) and egg yolk (5, 10 or 20%) concentrations than studied previously.

Spermatozoa incur significant damage during cooling and freezing as the lipids in the plasma membrane undergo a phase transition and ice crystals form within the cell^8^ with the majority of this damage occurring between −15 and −25 °C^15^. For this reason, the freezing rate must be a careful balance between the surface to volume ratio of the spermatozoa and the permeability of the membrane^16^, both of which are known to vary between species^17^. Alpaca spermatozoa have smaller heads than ram or bull spermatozoa^18^ and may therefore require a faster freeze rate than these species, though to date no studies have examined the effects of freezing and thawing rates on alpaca spermatozoa.

There are few AI studies in camelids, and results are generally poor compared with other production species in which AI and cryopreservation are used routinely. Whilst pregnancy rates of up to 73% using freshly ejaculated semen have been reported in alpacas^19^, a range of pregnancy rates between 0 and 70% have been reported (reviewed by Bravo, *et al*.^14^). These studies incorporated a wide range of sperm dose rates between 4 × 10^6^ ^20^ and whole, undiluted ejaculates (constituting up to 300 × 10^6^ spermatozoa^21^). Interestingly, insemination with dose rates of 4, 8 and 12 × 10^6^ spermatozoa Bravo, *et al*.^22^ achieved similar pregnancy rates of 53–67% to studies which used whole ejaculates (68%; Bravo, *et al*.^19^). These few studies and their highly variable results highlight the need for further study in this area, as the ideal dose rate and timing of insemination in alpacas remains unknown.

The ovulation time of female alpacas was recently defined as 26.2 ± 1.0 h after administration of an ovulation-inducing agent such as a GnRH agonist or β-NGF^23^. This knowledge allows precise timing of AI close to the time of ovulation to ensure that the spermatozoa are present in the female tract at the time of ovum release, thereby maximising the chance of fertilisation. This is especially important for cryopreserved spermatozoa which have decreased longevity due to freezing damage^15^.

Experiments 1–4 aimed to determine the optimal diluent type, storage method and concentration of egg yolk and glycerol for use in a semen diluent for ejaculated, papain-treated alpaca spermatozoa, as well as to determine the optimal freeze and thaw rates for use in a semen freezing protocol for this species. Experiment 5 aimed to test the *in vivo* fertility of papain-treated spermatozoa cryopreserved using the protocol developed in Experiments 1–4, using deep uterine AI, and additionally, to confirm the fertilising ability of papain-treated alpaca spermatozoa.

## Results

### Experiment 1: Egg yolk concentration and straw size

**Figure 1:**
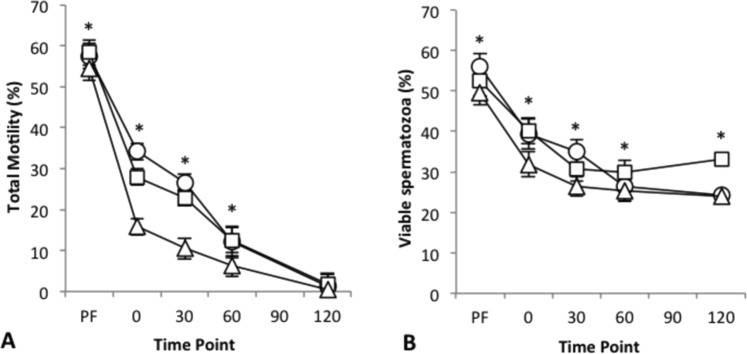
Mean ± s.e.m. total motility (**A**) and viability (**B**) over time (PF, pre-freeze and 0 to 120 min post-thaw) for 5% (○) 10% (□) and 20% (Δ) egg yolk concentrations. *Indicates significant difference among treatments at each time point (P < 0.05).

Motility differed among treatments and declined significantly over time (Fig. 1A; P < 0.001). Immediately post-thaw, spermatozoa frozen in medium containing 5% egg yolk had higher motility (34.17 ± 3.81%) than both 10% (27.92 ± 3.03%) and 20% egg yolk (15.83 ± 2.57%; P < 0.001), this trend continued until 60 min post thaw, when 20% egg yolk had significantly lower motility than the other two treatments. By 120 min, all three treatments had reduced to approximately 1% motility. Sperm viability was significantly higher at 5% (36.20 ± 3.51%) and 10% egg yolk (37.30 ± 3.42%) compared to 20% egg yolk (31.42 ± 2.82%; Fig. 1B; P < 0.001) and viability declined over time (P < 0.001). There was no significant difference between the two straw sizes in terms of motility (P = 0.47) or viability (P = 0.22).

### Experiment 2: Glycerol concentration

There was no significant difference among the spermatozoa frozen in the three glycerol concentrations in terms of mean motility (P = 0.11; 2%: 33.97 ± 1.36%; 5%: 32.67 ± 1.17%, 10%: 30.51 ± 1.66%) or viability (P = 0.10; 2%: 38.01 ± 0.90, 5%: 38.05 ± 0.40, 10%: 37.13 ± 1.13).

### Experiment 3: Diluent type and pellets vs. straws

**Figure 2:**
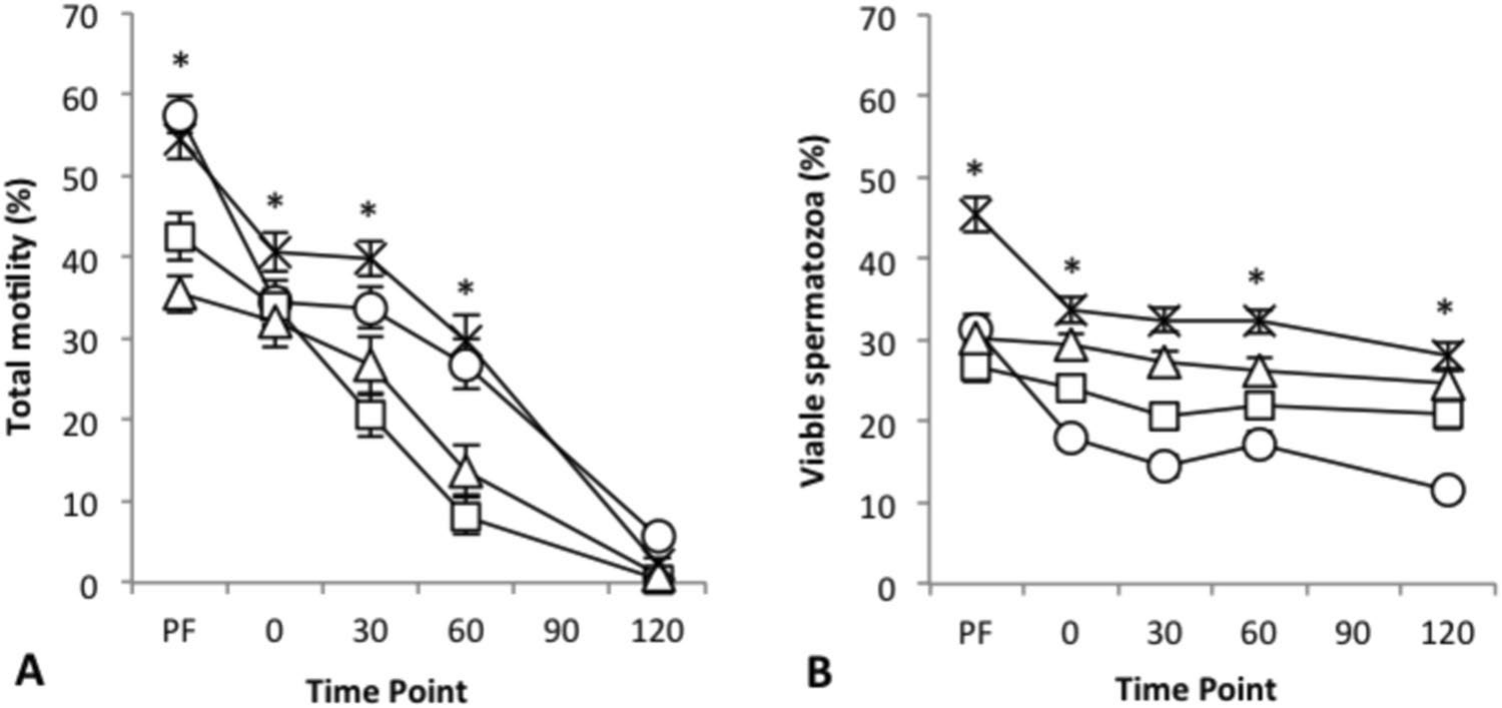
Mean ± s.e.m. total motility (**A**) and viability (**B**) among the four diluent types; SHOTOR (○); skim milk (□); INRA-96 (Δ); and lactose (×) over time (PF, pre-freeze and 0 to 120 min post-thaw). Means include pooled data for pellets and straws. *Indicates significant difference between treatments at each time point (P < 0.05).

Motility differed significantly over time and among spermatozoa frozen in each of the four diluent types (Fig. 2A; P < 0.001). At 0 min post-thaw, spermatozoa frozen in the lactose diluent had significantly higher mean motility than the other three diluent types (P < 0.001). At 30 and 60 min post thaw, there was no significant difference between lactose and SHOTOR and both had higher motility than skim milk and INRA-96. At 120 mins, motility had decreased significantly in all 4 treatments and no differences were found between the diluent types. Overall, post-thaw motility when pooled across time points was significantly higher when spermatozoa was stored in straws (24.24 ± 1.29%) compared with that frozen as pellets (20.12 ± 1.29%; P = 0.001) for all diluents.

Spermatozoa frozen in the lactose diluent had significantly higher viability than the other three treatments at all time points (Fig. 2B; P < 0.001). Additionally, spermatozoa frozen in straws had higher post-thaw viability (P < 0.001) when data from all post-thaw time points were pooled than spermatozoa frozen in pellets if diluted in lactose 34.22 ± 4.94% vs 27.25 ± 3.93%) or INRA-96™(29.48 ± 4.25% vs 24.40 ± 3.52%) but not if diluted in SHOTOR (14.70 ± 2.12% vs 15.83 ± 2.29%) or skim milk (23.88 ± 3.44% vs 20.20 ± 2.92%).

### Experiment 4: Freeze and thaw rates

There was no interaction in the post-thaw motility between freeze rate and time post thaw. When these data were pooled, spermatozoa frozen at 2 cm above liquid nitrogen (fast freeze; 23.41 ± 2.42) had higher post-thaw motility than that frozen at 4 cm (medium freeze; 20.75 ± 2.31) or 8 cm (slow freeze; 20.84 ± 2.39; P = 0.003) above liquid nitrogen.

When considering sperm viability, there was no interaction between freeze rates or time post-thaw. When these factors were dropped from the statistical model, the slow thaw rate had significantly higher viability than the fast thaw rate (36.88 ± 0.75 vs. 33.82 ± 1.03; P = 0.003).

### Experiment 5: Artificial insemination

At the time of insemination, 14/41 females had already ovulated and 27/41 were yet to ovulate. On Day 8, 15 of the 27 females yet to ovulate had low plasma progesterone concentrations (0.15 ± 0.06 ng.mL^−1^), indicating that they did not have an active CL and had not ovulated during the trial. These females were excluded from further analysis. Females with an active CL (26/41 females) had significantly higher plasma progesterone (4.09 ± 0.38 ng.mL^−1^) than the non-ovulators (P < 0.001; 0.15 ± 0.06 ng.mL^−1^).

Of those that had ovulated (n = 26) the pregnancy rate was 1/5 for fresh, 1/4 for fresh, papain-treated and 0/17 for papain-treated frozen-thawed spermatozoa, as detected by transrectal ultrasound between Days 19–26. There was no significant difference among the three treatments in terms of pregnancy rate (P = 0.10).

## Discussion

Methodical examination of different aspects of the cryopreservation protocol demonstrated that the highest post-thaw motility and viability of papain-treated, ejaculated alpaca spermatozoa was achieved using an 11% lactose diluent supplemented with final concentrations of 5% egg yolk and 2, 5 or 10% glycerol, loaded into 0.5 mL straws and frozen 2 cm above liquid nitrogen for 4 minutes, followed by thawing in a 37 °C water bath for 1 minute.

Previous studies in alpaca semen cryopreservation have yielded post-thaw motilities of <25% for both ejaculated^4,5^ and epididymal^11,24^ spermatozoa. By taking a systematic approach to the optimisation of a cryopreservation protocol for alpaca spermatozoa, the current study has achieved mean post-thaw motilities of up to 45% (lactose diluent, Experiment 3), a marked improvement of previously reported results in both epididymal and ejaculated spermatozoa (0%, Vaughan, *et al*.^25^; 12.5%, Morton, *et al*.^24^; 15–20%, Santiani, *et al*.^5^; 21.5%, Morton, *et al*.^26^).

In the current study, post-thaw motility and viability were highest when spermatozoa were frozen in lactose-based diluent, compared with skim milk, INRA-96™ or SHOTOR. Lactose was also superior to citrate- or tris-based diluents for the cryopreservation of epididymal spermatozoa^1^. Furthermore camel semen has been successfully diluted and stored using a lactose diluent^27,28^. In contrast, Bravo, *et al*.^20^ found that freezing ejaculated spermatozoa in a citrate-based diluent yielded post-thaw motilities of 30–40%, and a pregnancy rate of 26.3% when inseminated into 19 female alpacas, though details of their methods were not reported in sufficient detail to enable replication by others. Diluents containing glucose or fructose are used routinely in a number of species including sheep^29^ and cattle^30^ to dilute and store spermatozoa, as these sugars have a low molecular weight and can be metabolised by spermatozoa^31^. Lactose on the other hand, has a higher molecular weight and may not permeate the sperm plasma membrane, instead eliciting a protective effect from osmotic changes during dilution^29^. Additionally, alpaca seminal plasma contains negligible amounts of fructose compared to ram and bull seminal plasma and therefore the spermatozoa may not be able to metabolise this sugar at all^2^, this may explain why alpaca spermatozoa survive better in diluents containing lactose.

A study in Dromedary camels has utilised the milk-casein-based horse semen diluent INRA-96™ for semen preservation^32^. This diluent had not previously been tested on alpaca spermatozoa; though the success of previous studies in which alpaca spermatozoa was diluted in skim milk-based diluents^5,6^ suggested that it may be suitable. Interestingly, INRA-96 reacted poorly with papain, causing some agglutination of sperm heads, resulting in lower motility pre-freeze than the other three diluents and making it unsuitable for use under these conditions.

Morton, *et al*.^1^ found that epididymal alpaca spermatozoa had significantly higher post-thaw motility when frozen in pellets, compared with 0.25 or 0.5 mL straws (27.0 ± 5.2%, 8.6 ± 1.8%, 10.0 ± 2.4% respectively). Mamani-Mango, *et al*.^33^ also froze epididymal alpaca sperm using pellets and 0.25 mL straws and found that both motility (35.7 ± 16.5% vs 20.9 ± 12.3%) and viability (40.0 ± 13.6% vs. 30.4 ± 14.1%) was higher in pellets than straws. While these results are consistent with studies performed in other species, such as sheep^34^, the current study did not concur with this. Instead, the motility of spermatozoa frozen in straws was significantly higher than in pellets. Though the difference between these two storage methods is minimal, this finding will be of benefit for commercialisation of these techniques, as straws are more biosecure than pellets. In general, storage vessels with a higher surface-to-volume ratio, such as small volume straws, yield higher post thaw motilities than larger straws^35^, though no difference was found in the current study. Similarly, in boars^36^ and dogs^37^ spermatozoa frozen in 0.25 and 0.5 mL straws showed no difference in motility immediately post-thaw. Perhaps the small difference in surface-to-volume ratio between these methods is not enough to greatly affect sperm motility. This finding will be useful to operators performing AI in alpacas, as the larger 0.5 mL straws can hold greater numbers of spermatozoa, meaning less straws will need to be thawed per insemination dose.

There are no studies on the effects of freezing and thawing rates on ejaculated alpaca spermatozoa. In dogs, the height at which straws are frozen above liquid nitrogen (3.5 cm vs. 8 cm) has been found not to affect post-thaw motility, though the researchers did find an interaction between freezing rate and thawing rate, in that the slower freezing rate yielded the best results when combined with a rapid thaw rate^37^. No interaction between the freeze and thaw rates were found in the current study. Our results indicate that alpaca spermatozoa survive best when frozen at a relatively rapid rate and thawed at a slow rate (37 °C for 1 minute).

This study is the first to report a pregnancy using papain-treated alpaca spermatozoa and further supports the *in vitro* data obtained previously^3^ that demonstrates alpaca spermatozoa remain viable after papain-treatment for seminal viscosity reduction. However, no pregnancies resulted from insemination with frozen-thawed spermatozoa. As the dose rate used in the current study was higher than used in previous studies on fresh spermatozoa AI (50 × 10^6^ vs. 4–12 × 10^6^ used by Bravo, *et al*.^19^), and Experiments 1–4 had not indicated reduced sperm quality post thaw, this was unexpected. The *in vitro* experiments had assessed post-thaw sperm motility, plasma membrane and acrosome integrity to all be of acceptable levels, based on benchmarks used in other species^38^. Perhaps the reason for the lack of pregnancies resulting from AI with cryopreserved spermatozoa was due to some kind of freezing damage on a parameter that was not measured, such as oxidative stress, membrane potential and capacitation status. Consequently, further examination of papain-treated, cryopreserved alpaca spermatozoa is warranted to determine whether these spermatozoa are able to bind to and fertilise an oocyte *in vitro* (IVF), or whether the infertility is a result of impaired motility and inadequate transport once inseminated into the female tract. As pregnancy was achieved with fresh, papain-treated spermatozoa, at a similar rate to that achieved with fresh, untreated spermatozoa, we suggest that the enzyme treatment is not causing sperm damage resulting in infertility.

Alternative explanations for the overall low pregnancy rates may include incorrect timing of insemination with respect to ovulation, insufficient sperm dose, use of females of unknown fertility and/or incorrect semen placement during AI. Artificial insemination studies in alpacas are few, those that have been successful using fresh whole semen and fresh extended semen have resulted in pregnancy rates of between 39 and 68% when transcervical insemination was used^39^. The current study used high sperm doses (50 × 10^6^ motile spermatozoa). The pregnancy rates obtained by^22^ were higher with lower insemination doses of 4 (53.3% pregnancy rate), 8 (66.7%) and 12 × 10^6^ (61.5%) sperm. These rates are impressively high, when birth rates of 57–81% are considered normal for natural mating breeding programs in alpacas^40^. Future studies are warranted to investigate higher concentration doses, as the high volume of diluted semen used in the current study (up to 5 mL per dose) may have resulted in retrograde loss of spermatozoa post-insemination or dilution of important factors in the female tract that would normally facilitate sperm transport and fertilisation. Additionally, the timing of insemination should be further investigated. The optimal timing of insemination in camelids has not been determined, instead a narrow range of between 22 and 24 h post-induction have been used^25,41^. This study inseminated at 26 hours post-induction to be as close to the time of ovulation as possible^23^ based on the low longevity of cryopreserved spermatozoa and success of this timing in other spontaneously ovulating species^42,43^. Knowing the minimum dose rate and best timing of insemination required for fresh and papain-treated AI would greatly benefit the development of effective AI in this species.

This study has demonstrated that it is possible to freeze-thaw ejaculated, papain-treated alpaca spermatozoa and consistently achieve post-thaw *in vitro* sperm quality comparable with that seen in other species. It was also the first to obtain a pregnancy using papain-treated alpaca spermatozoa, highlighting the efficacy of papain-treatment for reducing alpaca semen viscosity. The lack of pregnancies obtained with cryopreserved spermatozoa emphasises the need for further studies on cryopreservation in this species, as well as the importance of the development of an AI protocol utilising fresh or papain-reduced semen to establish the optimum dose-rate, timing and site of insemination.

## Materials and Methods

This study used six male and 41 female alpacas under authorization from the University of Sydney Animal Ethics Committee (Approval numbers: 564 and N00/1-2012/3/5669). All experiments contained herein comply with the relevant Australian legislation including the Animal Research Act 1985, the Animal Research Regulation 2010 and the Australian code for the care and use of animals for scientific purposes. Experiments were performed using methods previously described in Kershaw-Young and Maxwell, 2012^4^. Males were >3 years of age with a body condition score ≥ 3 (out of 5), weighing ≥70 kg and with testes ≥3 cm in length. Females were >3 years of age (mean 96 ± 8 months) and were reproductively active (i.e. had a dominant follicle 6–10 mm in diameter at the time of buserelin administration).

### Experimental design

A series of five experiments were conducted. In the first four experiments, semen was collected from 3–4 male alpacas (3–4 ejaculates/male, n = 12) using an artificial vagina fitted inside a mannequin as described previously^44^. Within 5 min of collection, semen was assessed for volume, sperm motility and concentration as described below. Only samples with a volume >1 mL, motility ≥40% and sperm concentration ≥20 × 10^6^ spermatozoa/mL were used.

After collection, semen was diluted 1:1 with fraction A and treated with 0.1 mg/ml papain (final concentration; Sigma-Aldrich, St Louis, MO, USA) for 20 min at 37 °C. The activity of papain was halted by adding 10μM N-(trans-Epoxysuccinyl)-L-leucine 4-guanidinobutylamide (E-64; final concentration; Sigma-Aldrich, St Louis, MO, USA) at 37 °C for 5 min, this eliminated viscosity for all the ejaculates used. The diluted ejaculate was diluted further (2:1) with fraction B (fraction A supplemented with egg yolk), placed in a water jacket and cooled over 1.5 h to 5 °C. The cooled semen was further diluted (3:1) with fraction C (fraction B plus glycerol and Equex STM®; IMV Technologies, L’Aigle, France), to a final semen dilution rate of 1:3. The spermatozoa were allowed to equilibrate with fraction C at 5 °C for a further 30 min before being assessed for pre-freeze motility, acrosome integrity and viability, then loaded into straws and frozen above liquid nitrogen vapour or as pellets in indentations on dry ice and stored in liquid nitrogen, as described previously^38^. Straws were thawed by agitating in a 37 °C water bath before the semen was further diluted 2:5 with fraction A of the diluent (final dilution rate of original ejaculate 1:9) and incubated for two h in a 37 °C water bath. Pellets were thawed by placing them in a glass test tube in a 37 °C water bath while agitating the tube until the pellets liquefied. At 0, 30, 60 and 120 min post thaw, motility, acrosome integrity and viability were assessed.

### Experiment 1: Egg yolk concentration and straw size

In this experiment, fraction A consisted of 11% lactose and 3% BSA^45^. After papain treatment, the ejaculate was split into three equal parts and diluted with fraction B, supplemented with either 5, 10 or 20% (final concentration) egg yolk. After cooling, fraction C dilution consisted of matching egg yolk concentrations and 5% (final concentration) glycerol and 0.5% (final concentration) Equex STM®; fraction C). Semen was loaded into either 0.25 mL or 0.5 mL straws and frozen 4 cm over liquid nitrogen for 8 min or 10 min, respectively before being plunged into liquid nitrogen. Thawing of 0.25 mL straws was performed in a 37 °C water bath for 30 sec and 0.5 mL straws for 60 sec.

### Experiment 2: Glycerol concentration

Freezing was performed as per Experiment 1, except 5% egg yolk (final concentration) was used in fractions B and C. Semen was split into 3 aliquots after cooling (fraction B) and fraction C was supplemented with 2, 5 or 10% (final concentration) glycerol and 0.5% Equex STM®). Only 0.25 mL straws were used.

### Experiment 3: Diluent type and freezing method

In this experiment, the ejaculates were immediately divided into four aliquots after collection and diluted with fraction A that comprised of either lactose-based (11% lactose with 3% BSA), SHOTOR (214.6 mM tris, 4.2 mM citric acid, 66.6 mM glucose, and 49.9 mM fructose; Niasari-Naslaji *et al*. 2006b), INRA-96™ (IMV Technologies, L’Aigle, France) or skim milk (1% fat UHT skim milk supplemented with 270 mM fructose) diluent. Fractions B and C contained 5% egg yolk and Fraction C contained 5% glycerol and 0.5% Equex STM®. Each treatment was loaded into 0.5 mL straws and frozen for 10 min, 4 cm above liquid nitrogen vapour or as pellets in indentations on dry ice and stored in liquid nitrogen, as described previously^38^. Post-thaw analyses were performed over a 2 h incubation, as in the previous experiments.

### Experiment 4: Freeze/Thaw Rates

The optimal parameters identified from the previous three experiments were used for freezing. Fraction A: lactose diluent; fraction B: lactose diluent supplemented with 5% (final concentration) egg yolk; fraction C: lactose diluent supplemented with 5% egg yolk, 5% glycerol and 0.5% Equex STM®. Straws (0.5 mL) were frozen at three different heights above liquid nitrogen to vary the freeze rates; “slow” was 8 cm above for 12 min (average cooling rate of approximately −16.0 °C/min), “medium” was 4 cm above for 8 min (average cooling rate of approximately −23.9 °C/min) and “fast” was 2 cm above for 4 min (average cooling rate of approximately −47.8 °C/min), before plunging into liquid nitrogen for storage. Each of the three different freeze rates were thawed at two different rates; “fast thaw” in a 42 °C water bath for 20 sec (average thaw rate of approximately 699.0 °C/min) and “slow thaw” in a 37 °C water bath for 60 sec (average thaw rate of approximately 233 °C/min). Post-thaw analyses were performed over a 2 h incubation, as in the previous experiment.

### Experiment 5: Artificial insemination

Semen was collected and cryopreserved as described in Experiments 1–4 using the optimal diluent, cryoprotectant and freezing rate identified in these experiments. Semen for the fresh and papain-treated inseminations were collected as close to insemination time as possible and assessed for motility, concentration and volume. Each ejaculate was diluted 1:1 with fraction A of lactose diluent then split into two aliquots. One aliquot was treated with papain and E64 as described above, and the other was left untreated before being loaded into insemination pipettes (50 × 10^6^ motile spermatozoa/insemination dose; only ejaculates with ≥50% motility were used; volume ranged from 1–5 mL).

Each female was injected intramuscularly with 200 µg cloprostenol, a prostaglandin analogue (250 µg.mL^−1^; Estromil^®^; Troy Laboratories Australia, Glendenning, NSW, Australia) to regress any existing corpora lutea on the ovaries, followed by 4 µg buserelin, a GnRH agonist (4 µg.mL^−1^; Receptal^®^; MSD Animal Health Australia, Bendigo East, Vic., Australia) 24 h later to induce ovulation and synchronise the emergence of the next follicular wave as described by Kershaw-Young and Maxwell^4^. Luteolysis was induced 11 days after the induction of ovulation using 200 µg cloprostenol i.m.

Twenty-four hours after induction of luteolysis, the presence of a dominant follicle was determined by examining the ovaries via transrectal ultrasonography using a MyLab Five Vet scanner with a 7.5-MHz linear array transducer (Esaote Group, Genova, Italy)^4^. Females with a newly emerged dominant follicle (6–10 mm in diameter; n = 41) were injected intramuscularly with 4 µg buserelin and inseminated 26 hours later with 50 million motile sperm from one of three insemination treatments: 1) freshly collected spermatozoa (n = 7); 2) freshly collected papain-treated spermatozoa (n = 6); 3) papain-treated, frozen-thawed spermatozoa (n = 28). Each treatment was prepared and used as close to insemination time as possible. The insemination pipettes were guided through the cervix and into position via trans-rectal manual manipulation in the recumbent female. Semen was placed at the uterotubal junction ipsilateral to the site of ovulation by trans-cervical, deep intrauterine insemination. Pregnancy was diagnosed by observation of an amniotic sac via transrectal ultrasound on Day 19 or 25 post-insemination. Animals were monitored by a veterinarian for the duration of the study for any signs of an adverse reaction to the procedure.

### Analysis of sperm parameters

#### Concentration

Semen (10 µL) was diluted 1:9 in 90 µL 3% sodium chloride (Sigma) and sperm concentration was calculated using a haemocytometer^38^.

#### Motility

Sperm motility was assessed subjectively to the nearest 5% at 100 × magnification under phase contrast microscopy (Olympus, Tokyo, Japan) by placing 10 µL of semen on a warm slide and covering with a warm coverslip as described previously^38^. Camelid spermatozoa exhibit low mass motility, and tend to move in an oscillatory manner with limited progressive motility^21,46^. Consequently, all motile spermatozoa, whether oscillatory or progressive were considered motile and used to generate a value for total motility^26,47^.

#### Plasma membrane and acrosome integrity

Acrosome and viability were assessed using a dual stain method previously validated for formalin-fixed alpaca spermatozoa by Kershaw-Young *et al*.^3,4^. For each treatment at each time point, 30 µL of sample was fixed in 0.1% (final concentration) neutral buffered formalin and stored at 4 °C for a maximum of 12 h. The use of formaldehyde to fix and immobilise spermatozoa for subsequent acrosome analysis^48^ and plasma membrane analysis using propidium iodide has been described previously^4,49,50^. Semen (30 µL) was incubated with 6 µL fluorescent isothiocyanate-conjugated lectin from *Arachis hypogaea* (working concentration 40 µg/mL; FITC-PNA; Sigma) at 37 °C for 10 min. Next, 0.5 µL propidium iodide (working concentration 0.6 mM; PI; Molecular Probes, Eugene, OR, USA) was added to the sample and incubated at 37 °C for a further 5 min. Stained spermatozoa (20 µL) were placed onto a glass slide and covered with a 22 × 22 mm coverslip. A minimum of 200 spermatozoa were observed under phase contrast microscopy at 400× magnification using the Olympus BX51 fluorescent microscope with the U-MWIB filter (excitation filter 460–495 nm, emission filter 510–550 nm, 505 nm dichromatic mirror). Spermatozoa were considered to have non-intact plasma membranes if they stained red with PI, and acrosomes were considered not intact if the acrosome stained green with FITC-PNA. Spermatozoa that did not stain were considered viable (plasma membrane and acrosome-intact) and spermatozoa that stained red and/or green were considered non-viable, (plasma membrane and/or acrosome non-intact).

### Blood samples and hormone analysis

Jugular venous blood samples (5 mL) were collected on Day 8 and Day 15 post-insemination for determination of plasma progesterone concentrations. Samples were placed in heparinised tubes, centrifuged at 2,000 × *g* for 10 min and the plasma decanted and stored at −20 °C until assessment.

Plasma progesterone concentrations were determined using a commercially available double-antibody radioimmunoassay kit (Coat-a-Count^®^ Progesterone kit; Diagnostic Products, Los Angeles, CA, USA) previously validated in alpacas^4,51^. The sensitivity of the assay was 0.02 ng.mL^−1^ and the intraassay CV was 2.4%.

### Statistical analyses

Data were confirmed as normal through creation of a distribution plot. Data from Experiments 1–4 were compared for treatment differences over time using REML linear mixed model with a model reduction approach based on p-values. Experiment 5 data were analysed using Generalised Linear Mixed Models. Post hoc comparisons using the least significant difference (LSD) tests were used where appropriate. Data analyses were performed in Genstat v. 14 (VSN International, Hemel Hempstead, UK). For all analyses, P < 0.05 was considered significant. Data are presented as mean ± s.e.m.

## Data Availability

All data from these experiments are available on The University of Sydney’s escholarship repository.
